# Supplementary material for: Pervasive generation of non-canonical subgenomic RNAs by SARS-CoV-2
Source: Genome Med. 2020 Dec 1;12:108. doi: 10.1186/s13073-020-00802-w (PMC7704119; doi:10.1186/s13073-020-00802-w)
Supplement: Supplementary file 1 — Additional file 1: Figure S1. There is evidence of non-canonical junctions in diverse datasets. Figure S2. The landscape of non-canonical junctions across transcriptomes is generally consistent. Figure S3. Subgenomic RNAs containing non-canonical junctions are present in three independent dRNAseq datasets. Figure S4. There is elevated ORF1a coverage in CoV 229E. [file 13073_2020_802_MOESM1_ESM.pdf]

Figure S1

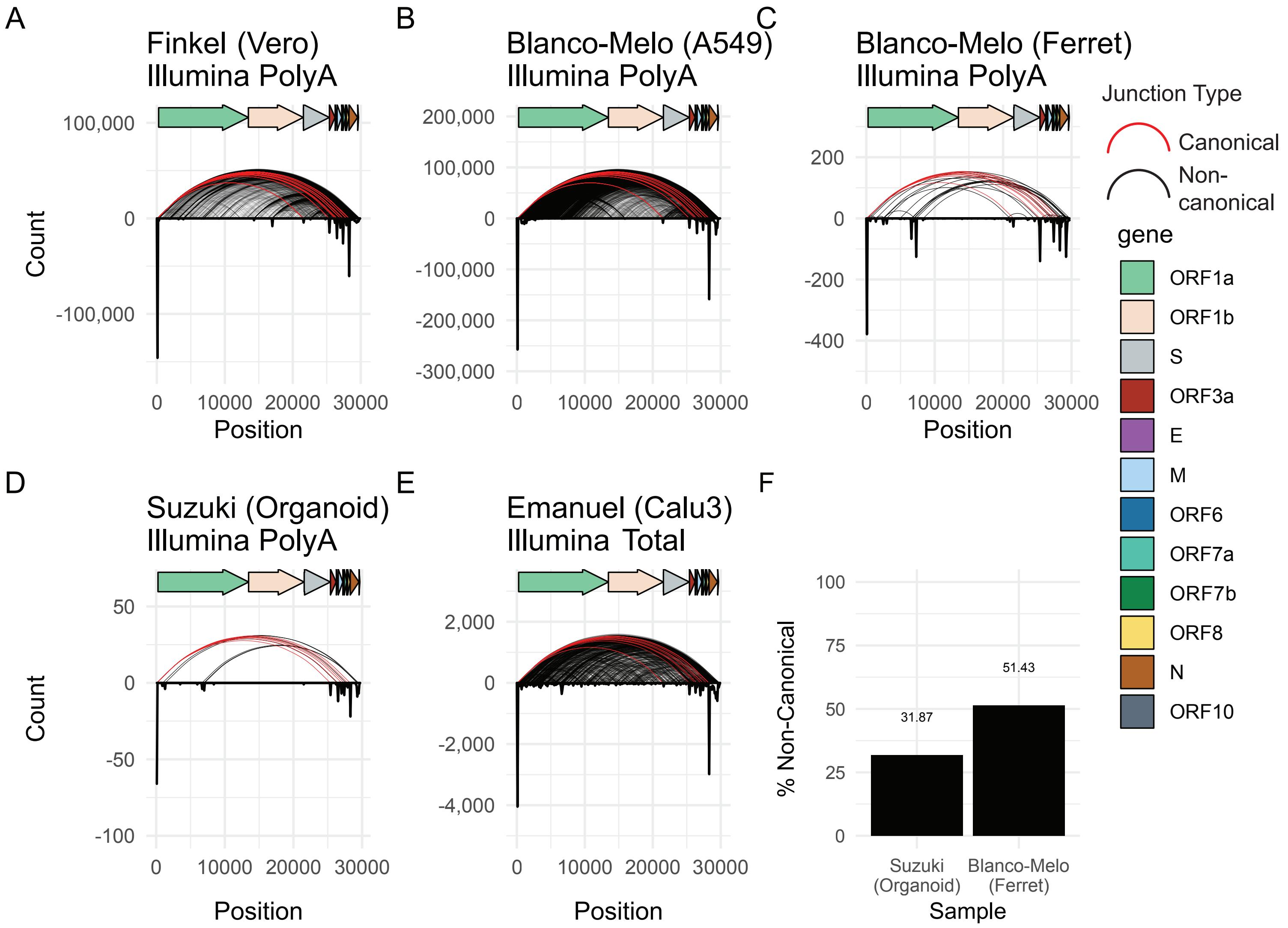

Figure S1. There is evidence of non-canonical junctions in diverse datasets.

A-E) For each location on the viral genome, a histogram of 5' and 3' junctions at that position was calculated and plotted as an inverse peak. The histogram bin size is 100 bases, meaning each inverse peak represents the cumulative count of 5' or 3' junctions occurring within that span. Curved lines represent the 5' and 3' locations of junctions that occur at least twice. Red curves represent canonical junctions, black curves represent non-canonical junctions.

F) The percentage of junctions that are canonical in the Suzuki (Bronchial organoids) and Blanco-Melo (Ferret) datasets was determined. Junctions were assigned as canonical if their 5' location was within 20 bases of the TRS-L and their 3' location within 15 bases of a TRS-B, and otherwise assigned as non-canonical.

Figure S2

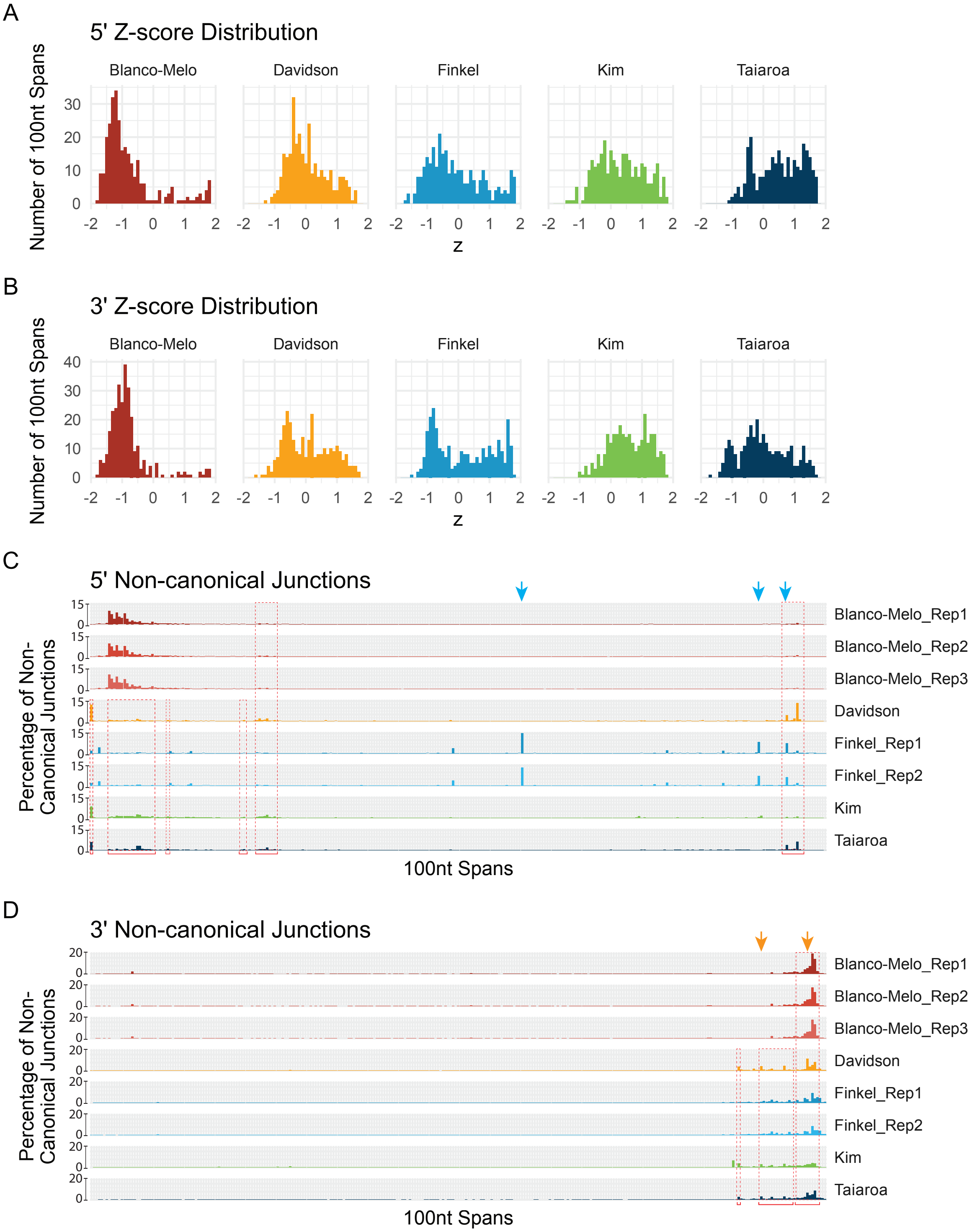

Figure S2. The landscape of non-canonical junctions across transcriptomes is generally consistent.

A-B) The percentage of non-canonical junctions with 5’ (A) or 3’ (B) ends in sequential 100nt spans of five transcriptomes (Blanco-Melo et al., Davidson et al., Finkel et al., Kim et al., and Taiaroa et al.) were determined, and Z-scores for each 100nt span were calculated. The Z-score distributions of the five datasets are plotted.

C-D) The percentage of non-canonical junctions with 5’ (C) or 3’ (D) ends in sequential 100nt spans across the entire SARS-CoV-2 genome were plotted from transcriptomes generated by Blanco-Melo et al., Davidson et al., Finkel et al., Kim et al., and Taiaroa et al. Here, replicate transcriptomes were included when available. Regions of similarity are boxed. Arrows indicate spans containing dataset-specific junction candidates identified in Figures 2C and 2D, and arrow color indicates the dataset in which the dataset-specific junction candidates are found.

Figure S3

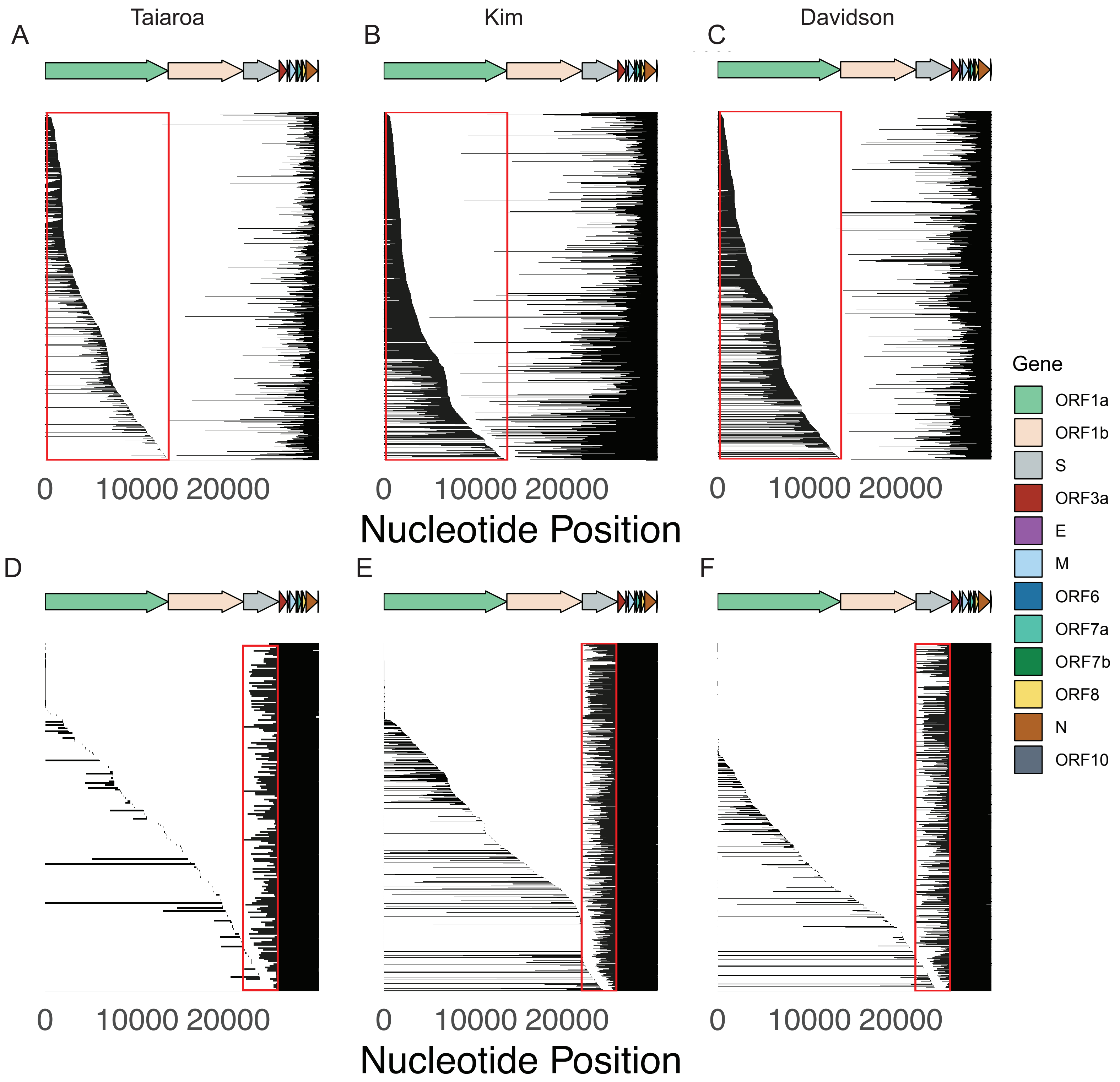

Figure S3. Subgenomic RNAs containing non-canonical junctions are present in three independent dRNAseq datasets.

A-F) Subgenomic RNAs with 5’ junctions occurring in ORF1a (A-C) or 3’ junctions occurring in S (D-F) are plotted for the three dRNAseq datasets. Each row is a read, and regions in black indicate the read contains that sequence.

Figure S4

A

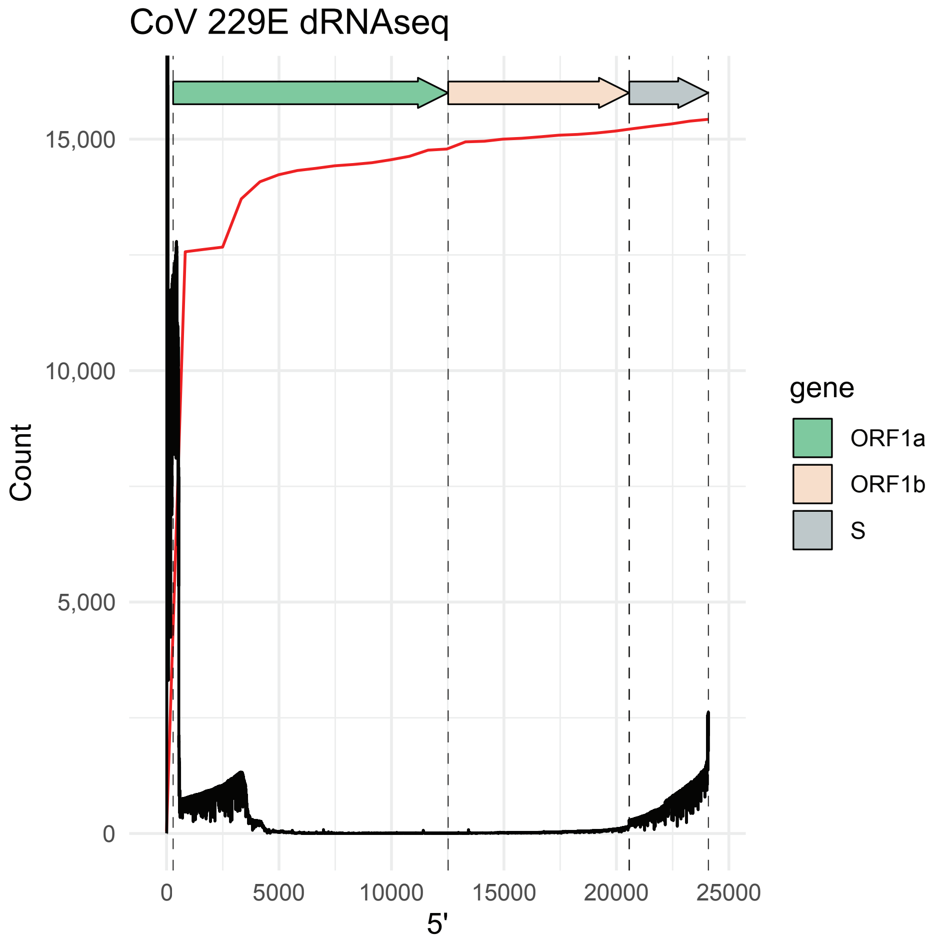

Figure S4. There is elevated ORF1a coverage in CoV 229E.

A) Read coverage (black) and cumulative 5’ junctions (red) are plotted for dRNAseq data from CoV 229E-infected cells.
